# Progestin Intrauterine Devices and Metformin: Endometrial Hyperplasia and Early Stage Endometrial Cancer Medical Management

**DOI:** 10.3390/healthcare5030030

**Published:** 2017-07-08

**Authors:** Oroma Nwanodi

**Affiliations:** Obstetrics and Gynecology Locum Tenens, Salinas, CA 93902, USA; o.nwanodi@juno.com; Tel.: +1-314-304-2946

**Keywords:** atypical endometrial hyperplasia, early stage endometrial cancer, endometrial cancer, endometrial hyperplasia, endometrial intraepithelial neoplasia, levonorgestrel intrauterine device, frameless IUD, metformin, mirena IUD, oral progestins

## Abstract

Globally, endometrial cancer is the sixth leading cause of female cancer-related deaths. Non-atypical endometrial hyperplasia (EH), has a lifetime progression rate to endometrial cancer ranging from less than 5%, if simple without atypia, to 40%, if complex with atypia. Site specific, long-acting intrauterine devices (IUDs) provide fertility sparing, progestin-based EH medical management. It is unclear which IUD is most beneficial, or if progesterone sensitizing metformin offers improved outcomes. For resolution, PubMed searches for “Mirena” or “Metformin,” “treatment,” “endometrial hyperplasia,” or “stage 1 endometrial cancer,” were performed, yielding 33 articles. Of these, 19 articles were included. The 60 mg high-dose frameless IUD/20 mcg levonorgestrel has achieved sustained regression of Grade 3 endometrial intraepithelial neoplasia for 14 years. Case series on early stage endometrial cancer (EC) treatment with IUDs have 75% or greater regression rates. For simple through complex EH with atypia, the 52 mg-IUD/10–20 mcg-LNG-14t has achieved 100% complete regression in 6-months. Clearly, IUDs have an outcome advantage over oral progestins. However, studies on metformin for EH, and of progestins or metformin for early stage EC management are underpowered, with inadequate dose ranges to achieve significant differences in, or optimal outcomes for, the treatment modalities. Therefore, outcomes from the feMMe trial for the 52 mg-IUD/10–20 mcg-LNG-14t and metformin will fill a gap in the literature.

## 1. Introduction

Globally, endometrial cancer (EC) is the fifth most common cancer in women, with about 320,000 incident cases in 2012, and the sixth leading cause of female cancer related deaths [1,2]. In the United States of America there were expected to be 60,050 EC cases and 10,470 EC deaths in 2016, making EC the fourth most common cancer in women, and the sixth leading cause of cancer-related deaths [3]. The European Union has about 88,068 cases and the United Kingdom about 7400 cases annually [4]. Endometrial hyperplasia (EH) progresses to EC at a rate of 1% if simple, 3% if complex, 8% if simple with atypia, and 29% if complex with atypia [5]. Non-atypical EH has less than a 5% lifetime progression risk for EC, but atypical EH or endometrial intraepithelial neoplasia (EIN), has up to a 40% lifetime risk of progression to EC [6]. As 84% of EC is Type I endometrioid adenocarcinoma, which has a stronger association with obesity than does non-endometrioid Type 2 EC, obesity with its concomitant increased estradiol and insulin levels, remains a risk factor for EC [7,8,9,10]. Endometrial cancer risk increases 1.6-fold for each 5 kg/m^2^ body mass index (BMI) increase [11]. Therefore, the global obesity epidemic is associated with increased EC and EH incidence [11]. Insulin resistance and non-insulin dependent diabetes mellitus (NIDDM) are also associated with EC: 36% of patients with EC are insulin resistant, and NIDDM has is associated with twice the incidence of EC than is non-diabetes [11]. 

Based on unopposed estrogen driving malignant endometrial proliferation, progestins have been the mainstay for fertility sparing medical management of EH and Stage I, grade I EC [7,8,12,13]. While oral progestin has historically been used for EH medical management, other delivery mechanisms are available. The 52 mg levonorgestrel (LNG) intrauterine device (IUD), which delivers 10 mcg to 20 mcg LNG daily for 5 years (52 mg-IUD/10–20 mcg-LNG-14t), the 60 mg LNG IUD, which delivers 14 mcg LNG daily for 5 years (60 mg-IUD/14 mcg-LNG), the 60 mg low-dose frameless LNG intrauterine system, which delivers 14 mcg daily for 3-years (60 mg-LD-frameless-IUD/14 mcg-LNG), and the 60 mcg high-dose frameless LNG intrauterine system, which delivers 20 mcg LNG daily (60 mg-HD-frameless-IUD/20 mcg-LNG), have the obvious advantage of long term patient adherence and proximity to the needed site of action [14,15,16]. In contrast to the 60 mg-IUD/14 mcg-LNG with a diffusion rate controlling ethylene vinyl acetate membrane [16], the 52 mg-IUD/10–20 mcg LNG-14t uses polydimethysiloxane in the drug reservoir, which may account for the difference in daily delivered dose. Selective progesterone receptor modulators (SPRMs) such as ulipristal acetate produce a distinct set of endometrial changes and have not been specifically trialed for EH treatment. Therefore, SPRMs were excluded from this review.

Metformin has been successfully used in a few cases of progestin nonresponsive atypical EH [17]. Cell line studies found that metformin is anti-proliferative to breast, endometrial, ovarian, and prostate cancers [11]. EC cell line studies demonstrated a dose dependent response to metformin [8]. Subsequently, meta-analysis indicated that breast, colorectal, hepatic, and pancreatic cancer incidence could be reduced by metformin [17]. A 985-patient retrospective cohort, 3.34-years median surveillance duration, found that metformin taking diabetics with Type 1 EC had greater overall survival than non-metformin taking diabetics and EC patients who did not have diabetes mellitus, *p* < 0.04, after adjusting for age, clinical stage, grade, chemotherapy treatment, radiation treatment and presence of hyperlipidemia [9]. With increasing metformin use in polycystic ovarian syndrome (PCOS) patients, an association between metformin and EC risk reduction became evident [13]. This was a significant finding as PCOS patients have a 4-fold greater risk of EC development than non-PCOS women [8]. Progestin resistance occurs in 30% of PCOS patients, therefore, alternative means of inhibiting estrogen-mediated endometrial proliferation are needed [8]. As metformin inhibits EC in cell line and population-based studies, it is plausible that metformin can treat EC precursor lesions ranging from simple hyperplasia to complex hyperplasia with atypia.

It will be shown that oral continuous or intermittent progestin has lower regression rates (higher progression rates), than do progestin containing IUDs. In other words, the locally- long-acting intrauterine progestin delivery provided by the 52 mg-IUD/10–20 mcg-LNG-14t, the 60 mg-IUD/14 mcg-LNG, the 60 mcg-HD-frameless-IUD/20 mcg-LNG and the 60 mcg-LD-frameless-IUD/14 mcg-LNG more effectively medically manages EH than does continuous or intermittent oral progestin. 

## 2. Materials and Methods 

**Figure 1:**
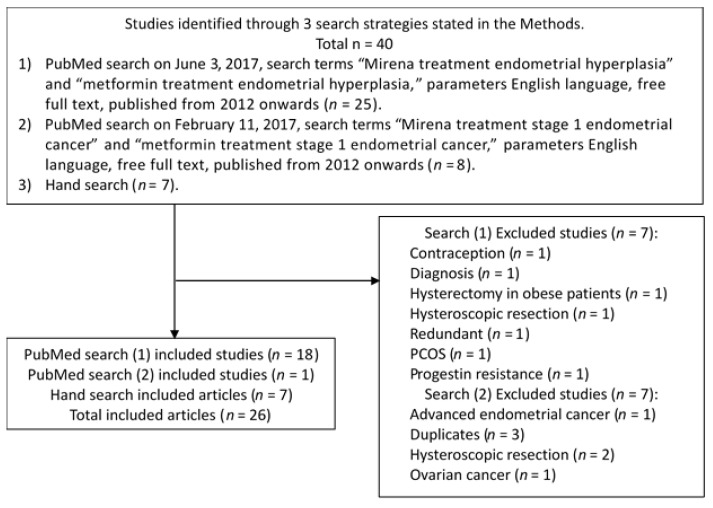
Article selection flowchart.

PubMed searches on June 3, 2017, search terms “Mirena treatment endometrial hyperplasia” and “metformin treatment endometrial hyperplasia” with the parameters English language, free full text, published from 2012 onwards yielded 25 articles. Of these, 1 was on contraception, 1 was redundant, 1 was concerned with diagnosis, 1 focused on PCOS, 1 focused on hysterectomy in obese patients, 1 included hysteroscopic resection, 1 explored progestin resistance, and 18 were included. PubMed searches on February 11, 2017, search terms “Mirena treatment stage 1 endometrial cancer” and “metformin treatment stage 1 endometrial cancer” with the parameters English language, free full text, published from 2012 onwards, yielded 8 articles. Of these, 3 were excluded as a duplicate, 2 were concerned with hysteroscopic resection as treatment or pretreatment followed by progestin treatment, 1 was concerned with ovarian cancer and 1 was concerned with advanced EC, leaving 1 inclusion. From the four PubMed searches, 19 articles were included, 14 articles were excluded. Hand search yielded seven additional included articles as shown in Figure 1. The included articles reviewed in the results section below are summarized in Table 1.

## 3. Results

### 3.1. Intrauterine Progestins

#### 3.1.1. Progestins’ Mechanism of Action 

Progestins modulate endometrial glands’ secretory differentiation, inhibit estrogen receptor function and endometrial cell mitosis, and are pro-apoptotic [7]. Progestins are also anti-angiogenic [7]. In vitro studies indicate that progestins stimulate stromal insulin-like growth factor binding protein-1 (IGFBP-1), which inhibits insulin-like growth factor-1 (IGF-1) expression and activity [7]. This is significant as IGF-1 is proliferative (anabolic) and anti-apoptotic, with increased expression in EH [7]. Thus, progestins inhibit as least two proliferative pathways. 

#### 3.1.2. Early Stage Endometrial Cancer Treatment: 52 mg-, 60 mg-, and 60 mg-frameless-IUDs

Grade 2 endometrial endometrioid adenocarcinoma in an 18-year old nullipara with Class III obesity, NIDDM and PCOS was treated with a 52 mg-IUD/10–20 mcg-LNG-14t [12]. Complete regression was noted on endometrial sampling at four 3-month intervals after 52 mg-IUD/10–20 mcg-LNG-14t placement [12]. A case series of 5 patients treated with medroxyprogesterone acetate (MPA) 500 mg daily concurrently with the 52 mg-IUD/10–20 mcg-LNG-14t showed 80% (4 out of 5 patients) complete remission without recurrence in 10.2 months mean surveillance period [7]. A single case of early stage EC treated with the 60 mg-IUD/14 mcg-LNG or 60 mcg-HD-frameless-IUD/20 mcg-LNG has been reported with sustained complete remission at an average of 32-months [16]. In one case series at 1-year post insertion, the discontinued 38 mg progesterone releasing IUD achieved complete regression of Stage IA, Grade 1 EC in six of eight (75%) women [7,12].

#### 3.1.3. Endometrial Hyperplasia Treatment: 52 mg-, 60 mg-, and 60 mg-frameless-IUDs

A multi-center randomized controlled trial (RCT) with 170 participants compared the 52 mg-IUD/10–20 mcg-LNG-14t to 10 mg MPA for 10 days monthly or continuously for EH treatment [18]. Pathologic diagnosis was based on the World Health Organization 1994 (WHO94) classification system and computer-based architectural and cytological features prediction of cancer (D-score) assessment [18]. Positive demonstration of 100% regression occurred at 6-months post treatment initiation when all participants in the 52 mg-IUD/10–20 mcg-LNG-14t arm with simple, complex, or complex hyperplasia with atypia, had normal endometrium [18]. However, cyclic progesterone was associated with 60% to 72% regression (69% overall), whereas continuous progesterone had an 88% to 100% regression rate [18].

For further surveillance, 153 participants from the above trial participated in a national, multicenter RCT of the 52 mg-IUD/10-20 mcg-LNG-14t compared to cyclic MPA 10 mg for 10 days per cycle, and continuous MPA 10 mg daily for 6-month’s treatment followed by reevaluation at 6, 12, 18, and 24 months [19]. Of the 153 participants, there were 18 non-responders, excluded from analysis. Of the remaining 135 participants, 55 subsequently relapsed (41%), with MPA recipients relapsing earlier than 52 mg-IUD/10-20 mcg-LNG-14t recipients [19]. Premenopausal participants were more likely to relapse than perimenopausal or postmenopausal participants, *p* = 0.001 [19]. Consistent with this, the higher the participant’s mean estrogen level, the higher the risk of relapse, *p* = 0.001 [19]. However, body mass index (BMI) was not associated with recurrence, *p* = 0.30 [19]. It is interesting that this study chose a 6-month treatment period, when the literature shows a 13.7% relapse rate if the 52 mg-IUD/10–20 mcg-LNG-14t is used for 5 years [19].

A RCT of 60 participants in total, compared EH treatment with the 52 mg-IUD/10–20 mcg-LNG-14t to MPA 10 mg orally daily for 12 days monthly for 3-months [20]. The 52 mg-IUD/10–20 mcg-LNG-14t achieved an 89.3% regression rate without any cases of disease progression, whereas cyclic MPA had a 70.4% regression rate with 7.4% disease progression [20]. While hirsutism occurred significantly more often in the MPA group, *p* = 0.013, this study, which did not use intent to treat analysis, was underpowered for the 18.9 percentage-point regression rate difference to achieve statistically significance [20].

At 1-year follow up, a randomized controlled trial (RCT) with 59 patients assigned to the 52 mg-IUD/10–20 mcg-LNG-14t versus 61 patients assigned to cyclic norethindrone (NET) 15 mg daily for 3-week cycles, indicated that the 52 mg-IUD/10–20 mcg-LNG-14t was more effective than cyclic NET medical management of non-atypical EH [7,21]. Intent to treat regression rate analysis of 3, 6, and 12-month surveillance pathology indicated the 52 mg-IUD/10–20 mcg-LNG-14t’s consistently better performance than cyclic NET: 67.8% versus 47.5%, relative risk (RR), 1.42; 79.7% versus 60.7%, RR, 1.31; and 88.1% versus 55.7%, RR, 1.58, respectively [21]. These findings are consistent with the literature, which shows 87.5 to 92% and 88.1% regression non-atypical or simple hyperplasia respectively for the 52 mg-IUD/10–20 mcg-LNG-14t versus 66% regression for oral progestins for non-atypical hyperplasia (*p* < 0.01) and 55.7% cyclic NET for simple hyperplasia respectively at 12 months [7,21].

A single case series of 20 women with non-atypical and atypical EH, achieving sustained, complete regression for an average of 32-months with the 60mg-IUD/14 mcg-LNG or 60 mg-HD-frameless-IUD/20 mcg-LNG has been reported [16]. A 44-year old woman with Grade 3 architectural atypia EIN was treated with a 60 mg-HD-frameless-IUD/20mcg-LNG from 2001 through 2015 [22]. From 2005 onwards the patient was amenorrheic, and from 2011 onwards endometrial sampling was interpreted as atrophic [22].

#### 3.1.4. Prophylaxis for Tamoxifen-Induced Endometrial Lesions: 52 mg-IUD/10–20 mcg-LNG-14t 

Meta-analysis of three RCT with a total of 359 participants found that the 52 mg-IUD/10–20 mcg-LNG-14t prevented tamoxifen-induced endometrial polyp formation, odds ratio (OR) 0.18, 95% confidence interval (CI): 0.13 to 1.02, *p* < 0.0001 [23]. Interestingly, the 52 mg-IUD/10–20 mcg-LNG-14t was associated with increased bleeding in tamoxifen users, (OR 6.20, 95% CI: 2.99 to 12.85, *p* < 0.00001), however, this was predominantly spotting which resolved in 1-year [23]. The 52 mg-IUD/10–20 mcg-LNG-14t did not prevent benign EH (without atypia), (OR 0.20, 95% CI 0.04 to 1.18, *p* = 0.08) [23]. The 52 mg-IUD/10–20 mcg-LNG-14t did not affect breast cancer recurrence, (OR 1.75, 95% CI: 0.64 to 4.80, *p* = 0.28), or cancer-induced death, (OR 1.22, 95% CI: 0.42 to 3.52, *p* = 0.71) [23].

#### 3.1.5. Limitations: 52 mg-IUD/10–20 mcg-LNG-14t 

Clearly the 52 mg-IUD/10–20 mcg-LNG-14t’s 1 in 1000 uterine perforation risk and invasive placement in the uterus are limitations that oral medications lack. Studies with IUDs cannot be completely blinded, and cannot have an innocuous a placebo control as other RCT can have. Normally an IUD string is left palpable in the vagina for IUD removal. The presence of the IUD string alerts the participant to the in utero IUD. Furthermore, any object placed in utero can exert a mass effect on the endometrium and uterus, therefore, a plastic IUD lacking hormonal or metallic active component is not inert. Incomplete participant and trial personnel blinding contributes to a performance bias [23]. The 52 mg-IUD/10–20 mcg-LNG-14t may also be associated with nausea and irregular vaginal bleeding even if EH or EC regression is achieved [12,21]. 

#### 3.1.6. Benefits: 52 mg-, 60 mg-, and Frameless-IUDs 

Clearly, the 52 mg-, 60 mg-, and frameless-IUD have long acting reservoir adherence advantages over daily oral progesterone for endometrial protection. While depo progestin precludes immediate treatment discontinuation, IUDs can be removed and progestin diffusion immediately halted. Oral progestin must be continuous to assure EH regression [18]. Nevertheless, oral progestin does not achieve endometrial concentrations as high as the 52 mg-IUD/10–20 mcg-LNG-14t [21]. Therefore, adherence is key to the success of oral progestin, and adherence is the default situation with IUDs. Medication adverse effects should also be considered. Oral and depo progestin administration can reduce tamoxifen's efficacy without preventing endometrial polyps, endometrial cyst, and leiomyoma formation [23]. Oral and depo progestin administration are associated with systemic adverse effects: Acne, cyclic breast symptoms, deep venous thrombosis, edema, fatigue, gastrointestinal symptoms including altered appetite and nausea, hirsutism, irritability, sleep dysfunction, and weight gain [12,18,20,21,23]. High doses of megestrol acetate (80 to 400 mg) or MPA (500 to 1000 mg) achieve endometrial response but have adherence reducing increased adverse effects [17]. Conversely, the 52 mg-IUD/10–20 mcg-LNG-14t is associated with one-third the incidence of adverse effects of cyclic or oral progestin treatment [18]. Lastly, while pain may be attributed to any treatment modality, pain is associated with nonresponsive EH [18].

### 3.2. Metformin for Endometrial Hyperplasia Treatment

#### 3.2.1. Metformin: Mechanism of Action 

Like progestins, metformin is anti-proliferative on the endometrium [11,13,17]. Metformin directly activates adenosine monophosphate (AMP)-activated protein Kinase (AMPK) via oxidative phosphorylation inhibition which reduces adenosine triphosphate (ATP). Metformin also promotes AMPK activation by liver kinase B1 (LBKI) [13]. AMPK activation decreases glyoxylase I expression and modulates the mTOR pathway, in turn sensitizing EC cell lines to cisplatin and paclitaxel [9]. Murine models have shown that AMPK activation inhibits cancer incidence [13].

Phosphoinositide 3-kinase (P13K) proto-oncogene serine/threonine protein kinase B (Akt) mammalian target of rapamycin (mTOR; P13K/Akt/mTOR) signaling pathway inhibition, mitogen-activated protein kinase (MAPK) inhibition, and glucose metabolism changes form part of metformin’s anti-proliferative effect [6,11]. Metformin blocks the epidermal growth factor signaling pathway, downregulates glyoxalase I expression, modulates the mammalian rapamycin pathway, and upregulates progesterone receptor (PR) expression, all of which are antiproliferative and progesterone sensitizing [8,17]. Metformin also chemosensitizes to progestins by down regulating the antioxidant transcription factor NF-E2-related factor 2 (Nrf2)/aldoketo reductase family 1 member C1 (AKR1C1) signal pathway of progestin-resistant endometrial epithelia [2]. By modulating NF-kB, MMP-2/9 Akt and Erk1/2 pathways, metformin reduces EC cell line invasion and metastasis [9]. Simultaneously, metformin induced lower insulin concentrations are anti-proliferative to insulin sensitive cancers [9]. Metformin and thiazolidinediones (TZDs) induce CGRRF1 expression, which is anti-proliferative to EC cells and upregulates caspase-3 dependent apoptosis [10].

Metformin upregulates GLUT4 mRNA and protein which are normally decreased in PCOS in comparison to non-PCOS [8]. Like progestins, metformin also inhibits the insulin/IGF-1 pathway, allowing metformin to be pro-apoptotic in uterine serous carcinoma [8]. Progestin withdrawal driven Nrf2/AKR1C1 down regulation permits unopposed estrogen driven endometrial proliferation. Metformin driven Nrf2/AKR1C1 down regulation does not depend on unopposed estrogen. In fact, metformin inhibition of testosterone provides less substrate for aromatase to convert into estradiol (E2), reducing the quantity of estrogen requiring progesterone balancing, thereby reducing endometrial stimulation. Metformin driven down regulation of glyoxalase I also reverses endometrial progestin resistance, increasing the efficiency of progesterone-estrogen balancing [2].

#### 3.2.2. Metformin: Single Agent

A case-controlled trial of 28 participants taking 850 mg twice daily for 7 to 30 days versus 12 participants not taking metformin found an average daily dose dependent 17.2% reduction in atypical EH and endometrioid EC Ki-67, 95% confidence interval (CI) −7.0%, −27.4%, *p* = 0.002 [11]. This study mirrors clinical practice as initial pathologic diagnosis was based on Pipelle endometrial biopsy specimens [11]. 

#### 3.2.3. Metformin with Cyproterone/Ethinyl Estradiol 2 mg/35 mcg

A case series of five insulin resistant PCOS patients diagnosed by the Rotterdam criteria, who had Stage 1A Grade 1 EC treated with metformin 1000 mg daily and Cyproterone/Ethinyl Estradiol 2 mg/35 mcg 21 days per month for 6 months achieved 100% complete regression [8]. This treatment regime had additional indirect benefits. Body mass index was reduced by 1.7 ± 0.85 kg/m^2^, *p* = 0.004 [8]. HOMA-IR was reduced by 0.48 ± 0.8, *p* = 0.25 [8]. Cyproterone is an anti-adrogenic progesterone agonist, therefore, not all combination oral contraceptive pills may have the same concurrent efficacy in EC treatment as Cyproterone/Ethinyl Estradiol 2 mg/35 mcg 21 days per month [8]. Cyproterone, chlormadinone, MPA, and megestrol acetate are all acetylated pregnane derivatives, whereas LNG and NET are testosterone-related progestins [24]. Therefore, it is biologically plausible that Cyproterone/Ethinyl Estradiol 2 mg/35 mcg and LNG containing IUDs will not have identical mechanistic effects on the endometrium 

#### 3.2.4. Metformin with Megestrol Acetate versus Single Agent Megestrol Acetate

A pilot study compared megestrol acetate 160 mg daily to metformin 500 mg trice daily with megestrol acetate 160 mg daily for treatment of atypical EH [17]. Only 16 of 30 participants, comprised of 8 metabolic syndrome patients and 8 patients at risk for metabolic syndrome completed the 12-week study [17]. Metabolic syndrome patients and those at risk for metabolic syndrome were evenly divided across both groups [17]. Endometrial response was unaffected by metabolic syndrome. Megestrol acetate alone achieved a 25% complete response at 12 weeks, whereas metformin with megestrol acetate achieved a 75% complete response rate [17]. This study is limited by the lack of intent to treat analysis. 

#### 3.2.5. Metformin: Limitations

Metformin use is limited by its gastrointestinal adverse effect profile. Metformin is associated with abdominal pain, diarrhea, lactic acidosis, nausea and vomiting, and taste changes [6]. Metformin decreases absorption of vitamins B9 and B12, in turn elevating homocysteine. Metformin is not for use in persons with moderate or worse renal function impairment.

## 4. Future Research

Consideration should be given to head-to-head trials of the 52 mg-IUD/10–20 mcg-LNG-14t and metformin combined with oral progesterone for EH and EC medical management. In vivo dose-escalation studies on metformin are needed to establish the optimal dose for EH treatment [11]. Metformin can also be studied expressly for EC primary and secondary prevention: in populations needing obesity and insulin resistance prevention, and in populations needing weight loss and insulin resistance treatment [11]. Use of the optimal metformin dose in subsequent studies will facilitate systematic reviews and meta-analysis. Conversely, use of the lowest megestrol dose only should be avoided, as dose dependent treatment effects do occur with megestrol [13]. To facilitate study outcome translation into clinical practice, studies should properly calculate the number needed to treat to find a significant treatment effect, if, in fact, there is a genuine treatment effect [8,13]. Intent to treat analysis should be used for all research studies.

Aromatase inhibitors that would reduce estrogen concentrations, gonadotrophin-releasing hormone agonists, and selective estrogen receptor modulators could be investigated for efficacy in preventing and/or treating EH and early stage EC [7]. GLUT1 and GLUT4 mRNA and protein expression in Type I EC could be studied in comparison to that in non-endometrioid Type 2 EC, as part of the underlying relationship between Type 1 EC, obesity, and response to metformin. While GLUT1 is over-expressed in EC, GLUT4 is over-expressed in breast, gastric, and lung cancers, and reduced in pancreatic cancer [25]. Thus, there is biologic plausibility beyond anti-proliferation mechanisms that supports metformin for treatment of EH and early stage EC. This, and the biologic effects of weight loss underlie the feMMe trial of the 52 mg-IUD/10–20 mcg-LNG-14t with/without metformin, with/without weight loss for early stage EC treatment in obese women [26]. The feMMe trial opened in Australia in 2012 [26]. Currently, feMMe trial results are awaited [26].

## 5. Conclusions

Progestins have least three anti-proliferative mechanisms: anti-angiogenesis, estrogen receptor inhibition and IGF-1 inhibition [7]. However, metformin has numerous anti-proliferative mechanisms: dual pathway AMPK activation, inhibition of the P13K/Akt/mTOR and EGF signaling pathways, and low insulin state induction [6,7,11,13,17]. Additionally, metformin increases progesterone receptor expression and chemosensitizes to progestins via the AKR1C1 signal pathway of progestin-resistant endometrial epithelia [2,8,17]. Therefore, metformin has the potential to increase progestin driven anti-proliferative mechanisms. 

Cyclic progestin has a 55.7 to 72% regression rate for EH, whereas continuous progestin has an 88 to 100% regression rate [7,18,21]. The 52 mg-IUD/10–20 mcg-LNG-14t has an 87.5 to 100% regression rate for EH, but does not prevent tamoxifen-associated EH incidence [7,18,20,21,23]. Of note, the 60 mg-IUD/14 mcg-LNG and the 60mcg-HD-frameless-IUD/20mcg-LNG have a 100% regression rate for EH [16]. Given relapse rates ranging from 13.7 to 27% for the 52 mg-IUD/10–20 mcg-LNG-14t and up to 45% for oral progestins, women on progestins for EH must never stop medical management with endometrial surveillance [19]. For complete remission of early stage EC, the 52 mg-IUD/10–20 mcg-LNG-14t may need to be used concurrently with metformin and/or oral progestins. To preclude a relapse, once childbearing is completed, surgical treatment for early stage EC should occur [7]. For the best outcomes, medical treatment of EH should require IUD use in 5- year increments [19]. Nonetheless, the 52 mg-, 60 mg-, and frameless-IUDs are more effective than continuous or cyclic oral progestins for medical treatment of EH and early stage EC.

Of note, with oral and intrauterine progestin therapy, premenopausal status and higher mean estrogen level are associated with EH recurrence, *p* = 0.001 [19]. Conversely, BMI was not associated with EH recurrence, *p* = 0.30 [19]. While blinding and placebo effect control can be achieved for oral progestin and metformin trials, the same is not easily said for IUD trials: Even if stringless IUDs are used, a placebo IUD could exert a biologic mass effect.

Hopefully, the feMMe trial, an international, phase II, RCT of the 52 mg-IUD/10–20 mcg-LNG-14t, metformin with the 52 mg-IUD/10–20 mcg-LNG-14t, and weight loss with metformin and the 52 mg-IUD/10–20 mcg-LNG-14t, for atypical EH and early stage EC patients will provide clarity on the effective of metformin concurrent with a progestin IUD [26]. It is biologically plausible that progesterone sensitizing metformin combined with any of the aforementioned locally long-acting IUDs will be more efficacious than metformin combined with orally administered continuous or cyclic progestins. However, until the feMMe trial results, confirmation thereof is lacking. 

If the feMMe trial supports metformin use with progestin IUDs for medical management of EH and early stage EC, a combined metformin and LNG IUD may become a reasonable treatment modality. A combined metformin–LNG IUD may also have contraceptive and antiglycemic benefits for female, reproductive age diabetics. However, as the feMMe trial excludes oral progestins, unanswered questions will remain after the feMMe trial. The comparative effectiveness of oral progestins combined with metformin versus that of the IUDs combined with metformin for EH or early stage EC medical management remains to be answered. 

## Figures and Tables

**Table 1:** Description of selected studies included in the review.

Source	Population	BMI	Diagnosis	Method	Treatment	Outcomes				
	Mean Age	(kg/m^2^)				3-Months	6-Months		12-Months	Other
[7]	5 - women	-	EC	-	52 mg-IUD/10–20 mcg-LNG-14t + MPA 500 mg daily	-	-	-		10.2 months: 80% remission
[8]	5- 29 y.o.	-	Stage 1A EC	Curettage	Diane-35 + metformin × 6 months	-	100% regression	-		-
[11]	28- 63.6 y.o.	35	Atypical EH, EC	Pipelle EMB	Metformin, 850 mg 2 × daily, × 20 days	-	-	-		17.2% reduced Ki-67 expression
[12]	18 y.o. P0	47.7	Grade 2 EAC	D&C	5yr-IUD	-	-	-		13-months: Disease-free
[13]	22- women	-	8- Simple EH, 9- DPE, 3- CH 2- low grade EC	-	Metformin, 500 mg 2 × daily	95.5% regression	-	-		-
[15,16]	21- 54 y.o.	-	12- simple EH 8- atypical EH 1-moderately differentiated EAC	Pipelle EMB or D&C	60 mg-LD-frameless-IUD/14 mcg-LNG × 3-years, then 60 mg-HD-frameless-IUD/20 mcg-LNG	-	-	-		10-year remission: 100%
[17]	8 women	-	Atypical EH	D&C	Metformin 500 mg 3 × daily + megestrol 160 mg daily	75% regression	-	-		-
[18]	53 women	-	6- simple EH 41- CH, 6- ACH	Pipelle EMB	52 mg-IUD/10–20 mcg-LNG-14t	-	100% regression	-		-
[19]	53 women	-	EH	Pipelle EMB	52 mg-IUD/10–20 mcg-LNG-14t × 6-months	-	-	-		2-year relapse: 41%
[20]	28- 38.3 ± 5.1 y.o.	26.5 ± 3.4	EH	Pipelle EMB	52 mg-IUD/10–20 mcg-LNG-14t	89.3%	-	-		Progression: 0
[21]	59- 45.2 ± 1.7 y.o.	31.6 ± 2.8	5- simple EH 54-complex EH	Hysteroscopy D&C	52 mg-IUD/10–20 mcg-LNG-14t	67.88% regression	79.7% regression	88.1% regression		Hysterectomy rate: 22%
[22]	44 y.o.	-	Grade 3 EIN	Hysteroscopy D&C	60 mg-IUD/14 mcg-LNG	-	-	-		12-years: Endometrial atrophy

ACH, atypical complex hyperplasia; CH, complex hyperplasia; D&C, Diane-35 (2 mg cyproterone acetate 35 μg ethinyl estradiol), Dilation and curettage; DPE, disordered proliferative endometrium; EAC, endometrial adenocarcinoma; EIN, endometrioid intraepithelial neoplasia; EH, endometrial hyperplasia; EMB, endometrial biopsy; IUD, intrauterine device; MPA, medroxyprogesterone acetate; P, para; y.o, years old; 52 mg-IUD/10–20 mcg-LNG-14t, 52 mg levonorgestrel (LNG) IUD delivering 10 to 20 mcg LNG daily for 5 years; 60 mg-IUD/14 mcg-LNG, 60 mg LNG IUD delivering 14 mcg LNG daily for 5 years; 60 mg-HD-frameless-IUD/20 mcg-LNG, 60 mg high-dose frameless LNG IUD delivering 20 mcg daily for 3 years; 60 mg-LD-frameless-IUD/14 mcg-LNG, 60 mg low-dose frameless LNG IUD delivering 14 mcg daily for 3-years.
